# Dentition in Infants

**Published:** 1893-05

**Authors:** 


### SELECTIONS.

### Dentition in Infants.

A communication from the pen of H. C. Wood, upholding gum lancing, and taking issue with the views of Forchheimer, on the subject, as detailed in his recent book on the Diseases of the Mouth, has been copied very generally by the medical press of the country. This extensive republication may fairly be taken as expressive of a general approval of the position taken by Prof. Wood.

Upon the question of difficult dentition and gum lancing, the medical world has been for some years divided; the smaller party taking the modern view that dentition is a normal process and rarely if ever produces dangerous symptoms; the larger party holding that dentition is responsible for most of the ills that infants suffer from, and that gum lancing is its sovereign remedy. This latter view is one of our most ancient possessions, having come down to us from Hippocrates. For centuries it remained unquestioned, and has consequently become firmly intrenched in both the professional and the lay mind.

John Hunter ascribed the following conditions to dentition: "Diarrhœa, costiveness, loss of appetite, eruptions on the skin, especially on the face and scalp, cough, shortness of breath, with a kind of convulsed respiration, and similar to that observed in whooping cough, spasms of particular parts either by intervals or continued, and increased and sometimes decreased secretion of urine, a discharge of matter from the penis, with difficulty in micturition, resembling symptoms of gonorrhœa in its violent form. The lymphatic glands are apt to swell at this time; if the child has a strong tendency to scrofula, this irritation will promote the disease. There may be many other symptoms with which we are not at all acquainted, the patients not being able to express their feelings."

Most, if not all these symptoms are attributed to teething today.

Perhaps the earliest opposition to these views was by Rosen von Rosenstein, in the middle of the last century; but his opposition was only partial. But Wichmann in 1800 expressed the true status of dentition when he said: "It is to be hoped that, in the future, dentition will be called up only when it would be impossible to comfort the relatives with the impotence to designate the true nature of the disease, or to quickly calm the laity."

Billard, who was a careful student of pathological anatomy, particularly with reference to infancy, found nothing to impress him with the importance of dentition.

As we look over the list of symptoms given by Hunter we find that many of them can be explained much more rationally by the results of modern positive observation. Diarrhœa has been shown to be intimately connected with fermentative processes; the respiratory symptoms are those commonly met in rickets, and in cases of pharyngeal adenoids; the convulsions are probably due to rickets; and the enlarged glands are tuberculous.

During the past summer this subject has occupied somewhat the attention of the Academy of Medicine of Paris. At the meeting of July 12, Magitot said "We wish that the so-called diseases of dentition, might be definitely erased from our medical nosology."

This brought a reply from M. Pamard on August 9, who took the following ground:

1. All difficult dentition is accompanied by a disturbance of the health of the infant.

2. In cold climates, and in cold seasons, all difficult dentition is accompanied by reflex phenomena on the part of the respiratory organs. In warm climates and in warm seasons, all difficult dentition is accompanied by reflex phenomena on the part of the digestive organs.

3. The diseases allied to dentition in the infant, pursue a course, and present characteristics, which are clearly defined and well established.

These propositions were supported only by the old argument of coincidence, but the essayist was upheld by MM. Le Roy de Mericourt, Herard, Charpentier, Peter, and Constantin Paul. He was opposed by MM. Ollivier, and Hardy.

In the study of this question, it is first necessary to separate dentition and gum-lancing. The first is a possible pathological condition, while the second is a therapeutic procedure.

We think it can be said without fear of contradiction, that there is not a single positive observation which has ever been recorded to prove that dentition produces general or reflex symptoms. It is undeniable that at the period in life when dentition is in progress, the infant is subject to certain disorders which occur much more commonly than at any other period in life.

If it could be shown that dentition was the only peculiarity of the infant, then its causative influence would be clear. But dentition is not the only peculiarity of the infant, and co-existing phenomena can only be classed as coincident. The most profound characteristic of infancy is that it is the period of most rapid growth and development of all organs; and careful observation of infants reveals numerous and great deviations from the normal growth and development in many instances. It will probably not be denied that such deviations are found most commonly in infants who have been artificially fed. In infants improperly fed, and this term is too extensive to attempt to define here, reflex manifestations are very readily produced, and it is not improbable that even a normally developing tooth may, in such an infant, be the exciting cause of trouble. We have seen infants, who would invariably have a bronchial attack immediately before the proruption of a tooth, but they have invariably been infants who were suffering from demonstrable deviations from normal nutrition. We have further found that after improving the nutrition of these infants, the further progress of dentition was unaccompanied by symptoms.

In such cases while it would be just as well perhaps to recognize the possible influence of dentition, its subordinate importance should be kept clearly in view. The great danger of teething is in the diagnosis. for when this is once made, the important underlying conditions are apt to be neglected, and permitted to progress to the death of the child.

Dentition is a convenient scapegoat, and Ollivier has well said in the discussion just referred to: "During the nearly ten years that I have been connected with the Hospital for Sick Children, it has often happened that children brought to me for diseases of this type (teething) have been found to be suffering with an altogether different affection. It is very easy to invoke this diagnosis, but by passing in review the different organs and apparatuses, the diagnosis can easily be rectified."

But if dentition cannot be shown to be the great etiological factor of infantile disorders, it does not follow that gum lancing should be abandoned. It is difficult to overlook the numerous instances in which careful observers have thought they have obtained good results from its use, but it would be well also to bear in mind the many cases in which it has failed. As a therapeutic procedure it may have some value, but the indications for its use must be sought elsewhere than in a supposititious condition of teething. We should like to offer the following conclusions:

1. Before the diagnosis of "teething" is made, there should first be carefully excluded, organic disease of all organs, infection, intoxication, and perversion of nutrition.

2. Gum lancing as a therapeutic measure should stand on its own merits, and be studied apart from any supposititious and undemonstrable process of teething.

